# 

**Published:** 1911-07-15

**Authors:** 


From the sixteenth report of the Board of Health of the town of Montclair, in New Jersey, from January 1, 1910, to December 31, 1910, we abstract the following points: The birth, marriage, general death-rates, and the death rate from communicable diseases of this town for the year 1910 were respectively 194, 7.3, 11.1, and 4.0 per 1,000 inhabitants living, as compared with rates respectively of 20.7, 7.2, 10.1, and 2.3 for the previous year. The death rate per 100,000 living from phthisis was 138, and from all forms of tuberculosis 179, giving percentages of 12.4 and 16.1 with regard to deaths from all causes. In the previous year these rates per 100,000 of population had been 72 and 92 respectively, with percentages of the total deaths of 7.1 and 9.0 respectively. The most unsatisfactory features of the report are those in connection with tuberculous disease and the prevalence of scarlet fever. The epidemic of this last disease was fortunately of a mild type, there being only three deaths out of 163 cases reported. Diphtheria cases showed only a slight increase, whereas those due to typhoid fever and measles exhibited a marked falling off in numbers. There was no fatal case of typhoid fever reported. The birth rate recorded is the lowest rate for over twenty years. The infantile death-rate of 111 per 1,000 births is the highest recorded since 1905. Bacteriological examinations undertaken at monthly intervals show that the town is possessed of a good water-supply. The authorities continue alive to the importance of a good milk-supply, and the herds are subjected to the tuberculin test. All milk has to be delivered in bottles, and those who from time to time endeavour to sell "dip" milk are smartly fined. The report contains figures regarding the bacteriological tests with regard to, and percentage of solids in, the samples collected from the eleven milk dealers in the town.

### BUILDINGS CONTEMPLATED AND IN PROGRESS.

Andover.—For the building of an isolation hospital at Andover a tender of £1,700 by Messrs. Beale and Sons has been accepted.

Biggleswade.—For alterations and additions to the Workhouse Infirmary, and for which Mr. Walter Jackson is architect, a tender of £2,709 by Mr. Redhouse, of Stotfold, has been accepted.

Billericay.—Tenders have now been received for the erection of a Nurses' Home on the workhouse premises. The highest is £850; the lowest, £693, has been accepted. The wide tendering may be accounted for by the fact that builders had to take off their own quantities. The approximate price given to the Board of Guardians by Mr. Hugo Bird, architect, was £770.

Brighton.—It is proposed to erect four receiving homes and a hospital adjoining Warren Farm. Particulars may be had from the Clerk to the Board of Guardians, Mr. B. Burrfield.

Bristol.—Additional workhouse accommodation is projected, at a cost of about £27,000, by the Bristol Board of Guardians.

Cannock.—Mr. Herbert Whitehead is architect for the alterations and additions to the Cottage Homes. The lowest estimate of eight received is £499 10s.; the highest £611.

Chipping Norton.—Mr. J. R. Wilkins, of Oxford, is architect for the rebuilding at Chipping Norton Workhouse, recently destroyed by fire. Tenders are now invited.

Colchester.—For extensions to the infirmary, at a cost of £1,900, a tender by Mr. G. Beaumont, of Lexden, has been accepted.

Downpatrick.—Mr. Fitzsimons, builder, Ballynarry, has submitted a successful tender of £960 for the erection of a dispensary and residence at Downpatrick.

Goole.—Messrs. Brodrick, Lowther, and Walker, of Hull, are architects for the building of a doctor's residence at Goole estimated to cost £2,500.

Hackney.—The Guardians invite tenders for the erection and completion of a nurses' home and lunacy wards at the Homerton Infirmary. The form of bond and plans may be seen at the Guardians' offices, and tenders are to be delivered before July 19.

Llandudno.—The new Convalescent Home at Llandudno is being erected from a design by Mr. J. T. Brealey, of Hanley. It will cost about £5,000.

### MEDICAL APPOINTMENTS.

Dr. Philip A. Harry, M.D., D.P.H., late resident ophthalmic officer at the Leeds General Infirmary, has been appointed hon. ophthalmic surgeon to the Rochdale Infirmary.

Dr. W. M. Munby, late ophthalmic and aural house surgeon at Leeds General Infirmary and of the Central London Throat Hospital, has been appointed Dr. Harry's successor.

The following appointments have been made at Guy's Hospital: To be consulting dental surgeon, Mr. W. A. Maggs; to be dental surgeon, Mr. J. B. Parfitt, L.R.C.P., M.R.C.S., L.D.S. Eng.; to be assistant dental surgeon, Mr. H. P. Aubrey, L.R.C.P., M.R.C.S., L.D.S. Eng; to be assistant surgeon, Mr. E. C. Hughes, M.A., M.C. Cantab., F.R.C.S. Eng.

### GIFTS.

Miss Eliza Wood, of Lee, Kent, has left £100 each to the Hospital for Incurables at Putney and to the Earlswood Asylum for Idiots.

The proceeds of the matinée organised by Sir Charles Wyndham in aid of the Ladies' Association Convalescent Fund of the City of London Hospital realised £702.

Mrs. Mary Ann Routledge, of York, has left £1,200 to the York County Hospital and £200 to the York Dispensary. The residue of her estate she left to the York County Hospital.

As a result of the recent sale opened by the Duchess of Norfolk at Ennismore Gardens, S.W., the Ladies' Association has handed over £638 towards the Endowment Fund of University College Hospital.

Mr. James Clason Harvie (formerly James Harvie Clason), of Edinburgh and Lanarkshire, left £3,000 each to the Edinburgh Royal Infirmary and the Glasgow Royal Infirmary, Scotland; and £1,000 to the Royal Hospital for Sick Children, Glasgow.

Prince Alexander of Teck has received for the Prince Francis of Teck Memorial Fund £165 odd from Selfridge's and £18 odd from Waring and Gillows, the proceeds of the sale of seats, together with £11 odd collected from the guests of D. H. Evans and Co. on the occasion of the Royal progress through London.

Mr. Henry Kirk, of Beeston, Nottingham, lace manufacturer, has left £5,000 upon trust to pay the income to the Nottingham General Hospital; £1,000 to the Nottingham General Dispensary, Broad Street; £300 each to the Nottingham Children's Hospital, the Nottingham Hospital for Women, Castlegate, the Mablethorpe Convalescent Home, and the Skegness Convalescent Home; and £200 to the Nottingham Eye Infirmary, St. James's Street.

Miss Anna Thorne Harris, of Plymouth, has bequeathed £2,000 to the South Devon and East Cornwall Hospital at Plymouth; £1,000 to the South Devon and Cornwall Institution for the Blind at Plymouth; £500 each to the Plymouth Royal Eye Infirmary and the Plymouth Public Dispensary. The residue of her estate she left in equal shares to the South Devon and East Cornwall Hospital at Plymouth, the Devon and Cornwall Sanatorium for Consumptives at Didworthy, South Brent, and to one other purpose.

### JOTTINGS.

The Coalville and District Charity Football Association has sent £30 to the Leicester Infirmary to furnish a sitting-room in the Nurses' Home.

Miss Jessie Matheson has sent £25 to provide some little treat for the nurses of the Cancer Hospital to commemorate the Coronation.

As the result of the recent matinée at the Leicester Palace Theatre a sum of nearly £94 has been handed to the Leicester Infirmary and Children's Hospital, and the Board have had pleasure in submitting the names of Mr. Oswald Stoll (managing director of the theatre) and Mr. Arthur Baum (the local hon. treasurer of the matinée) for election to life governorships.

A new balcony, measuring thirty-six feet long and six feet wide, has been presented and built for the children's Victoria ward at the Dover Hospital. The wall on to which the balcony abuts bears a brass plate with the words, "This balcony was presented to the hospital for the children's benefit by Mrs. Arthur Burr. June 1911." The cost of the balcony was £142.

### APPOINTMENTS VACANT.

Full particulars of these vacancies can be obtained on reference to our advertisement columns :

Cardiff Union.—Assistant Medical Officer.
City of Birmingham Education Committee.—Medical Officer.
Coventry and Warwickshire Hospital.—Junior House Surgeon.
Great Northern Central Hospital.—Resident Medical Officer.
Royal Albert Hospital, Devonport.—Assistant House Surgeon.
Royal Surrey County Hospital, Guildford.—Assistant House Surgeon.
Wolverhampton General Hospital.—House Surgeon.

### COMING EVENTS OF THE WEEK.

[Communications for this column must be sent to 29 Southampton Street, Strand, before noon on Wednesday.]

Saturday, July 15 (to-day).—The annual View Day at the Brompton Hospital Sanatorium at Frimley. Lord Cheylesmore, chairman of the Committee of Management, invites all who are interested. There will be a special train for visitors, and vouchers for tickets at reduced rates may be obtained from the Medical Superintendent at Frimley.

Princess Christian opens the new King Edward Memorial Hospital, Matlock Lane, Ealing, 4 p.m.

